# Economic Burden and Quality of Life of Hepatocellular Carcinoma in Greater China: A Systematic Review

**DOI:** 10.3389/fpubh.2022.801981

**Published:** 2022-04-21

**Authors:** Huimin Zou, Meng Li, Qing Lei, Zejun Luo, Yan Xue, Dongning Yao, Yunfeng Lai, Carolina Oi Lam Ung, Hao Hu

**Affiliations:** ^1^State Key Laboratory of Quality Research in Chinese Medicine, Institute of Chinese Medical Sciences, University of Macau, Macau, Macao SAR, China; ^2^School of Public Health and Management, Guangzhou University of Chinese Medicine, Guangzhou, China; ^3^Department of Public Health and Medicinal Administration, Faculty of Health Sciences, University of Macau, Macau, Macao SAR, China

**Keywords:** hepatocellular carcinoma, economic burden, quality of life, systematic review, China

## Abstract

**Background:**

Hepatocellular carcinoma (HCC) accounts for more than 85%-90% of primary liver cancer globally, and approximately 45% of deaths from HCC occur in greater China. This disease poses a significant economic burden for patients, payers and society and significantly affects patients' quality of life (QoL). However, such impact of HCC in greater China has not been well characterized. This review was conducted to analyze the current evidence about the economic and humanistic impact of HCC in greater China for informing national disease management and identifying clinical gaps yet to be resolved.

**Methods:**

A systematic search literature using seven databases (Web of Science, PubMed, Medline, Cochrane Central, China National Knowledge Infrastructure, Wanfang, and Weipu) was performed to identify interventional and observational studies that reported the impact of HCC on cost or QoL and published before April 6, 2021. The focus population included adult patients with HCC in greater China. This review excluded any studies that focused on any specific treatment. Study quality was assessed using the Effective Public Health Practice Project tool.

**Results:**

Of 39,930 studies retrieved, 27 were deemed eligible for inclusion. The methodologies, perspectives and data sources used in studies were heterogeneous. In greater China, while few studies reported the health expenditures of HCC patients and investigations about economic burden at national level was lacking, the significant economic impact of HCC on patients and their families had been reported. Health-related costs increased as the disease deteriorated. Additionally, HCC also has a negative impact on the QoL of patients, mostly in terms of physical, cognitive, social functioning and severe symptoms.

**Conclusions:**

HCC has brought significant economic and QoL burden to patients in greater China. Both physical and psychological factors predicted QoL in patients with HCC in greater China. Future studies should explore the disease-related economic effects on Chinese patients and their families, the effects of physical and psychological factors on QoL and the relationships of physical and psychological factors in the region.

**Systematic Review Registration:** www.crd.york.ac.uk/prospero/display_record.php?RecordID=278421, PROSPERO: CRD42021278421

## Introduction

Hepatocellular carcinoma (HCC) is the major pathological classification of primary liver cancer, accounting for 85%-90% liver cancer globally (1). In terms of global incidence, HCC ranks 7th, and there are about 850 thousand new cases of HCC worldwide every year (1, 2). The incidence of HCC increases with age and is 2.7 times higher in males than in females (3). HCC is a highly fatal cancer, causing approximately 800 thousand deaths every year (4). It is the fourth leading cause of cancer-related deaths, second only to lung cancer, colorectal cancer, and gastric cancer (5). The high prevalence of HCC poses increasing demand for treatment and hospitalization, which bring huge disease burden for both patients and the society (6). In greater China, HCC is the fourth highest incidence of malignant tumors, with 466 thousand new cases each year accounting for about 50% of the total global new cases (7, 8); there are about 444 thousand deaths due to HCC each year accounting for approximately 45% of the total deaths from HCC worldwide.

HCC can be induced by multiple etiologies and risk factors such as Hepatitis B Virus (HBV) infection, Hepatitis C Virus (HCV) infection, alcohol abuse, metabolic liver disease (such as non-alcoholic fatty liver disease) and exposure to dietary toxins (such as aflatoxin and aristolochic acid) (9). Among them, chronic hepatitis caused by viral infection accounts for more than 70% of HCC in the world (10). Chronic hepatitis B (HBV) and hepatitis C (HCV), as well as aflatoxin, are classified as Group 1 carcinogen by the International Agency for Research on Cancer (IARC) (11, 12). Recent studies suggested that patients with diabetes, obesity, smoking, drug-induced liver damage and men over the age of 40 also carry a higher risk of HCC (13, 14). HCC may be developed by the additive effects of multiple complex pathogeneses.

Most HCC cases were discovered at a late stage, and patients suffered from poor prognosis and short survival due to limited treatment options during advanced stage (15–17). The treatment of HCC can be divided into two types: surgical and non-surgical. Surgical treatment includes curative resection, liver transplantation, etc.; non-surgical treatment mainly includes radiofrequency ablation, trans-arterial chemoembolization (TACE), radioembolization, and systemic targeted agent, etc (7, 18). The utilization of medical, clinical treatments and work loss due to the disease brings a huge negative impact on the economic status and quality of life (QoL) for patients with HCC and their families.

Research showed that the overall financial charges for HCC hospitalization, especially for the costs related to disease progression, increased substantially in the past decades (6, 19, 20). The total inpatient expenses related to HCC increased from 1.0 billion USD in 2005 to 2.0 billion USD in 2009 in USA (19). In terms of QoL, extrahepatic manifestations and complications largely affected physical, social, emotional, and functional well-being of patients with HCC (21, 22). Understanding the economic impact and QoL affected by HCC is essential to determine the factual disease burden on patients and their families. Despite that greater China has a high prevalence rate of HCC in the world and has developed relevant policies to reduce patients' burden, the evidence about the economic burden and the QoL in patients affected by HCC in greater China has not been well characterized.

Therefore, a systematic review of the published literature on the economic burden and QoL in patients with HCC in greater China was conducted to provide evidence for public health strategies of HCC management and identify clinical gaps to inform future research of HCC in greater China.

## Methods

The reporting of this systematic review was performed according to the PRISMA statement (23). The protocol of this systematic review was registered with PROSPERO (CRD42021278421).

### Eligibility Criteria

Eligibility criteria presented in Table 1 were specified according to the PICOS approach (24). Interventional and observational studies published in English or Chinese were included if they reported direct and indirect costs or QoL measures based on clinical instruments. Both prospective and retrospective studies were included. The duration of follow-up or the year of publication was not restricted. The focus population included adult patients suffering from any stage of HCC in greater China. This review excluded any studies that focused on any specific treatment. Commentaries, conference abstracts, reviews, editorials, letters, case reports, protocols, *in vitro* studies, animal studies, and data-uncompleted publications were also excluded. Included studies were further grouped into economic burden studies and QoL studies.

**Table 1:** Eligibility criteria.

**Inclusion criteria**	
Patient population	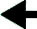 Adults (age ≥ 18 years) with any stage of HCC in greater China
Intervention	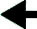 All interventions except for any specific treatment
	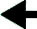 No intervention
Comparator	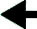 All interventions except for any specific treatment Baseline
	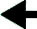 No comparator
Outcomes	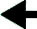 Direct costs Indirect costs
	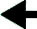 Health insurance payments QoL measures based on clinical instruments
Study type	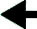 Interventional studies
	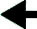 Observational studies
Language	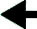 English Chinese
**Exclusion criteria**	
Publication type	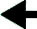 Commentaries
	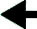 Conference abstracts
	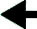 Reviews Editorials
	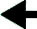 Letters
	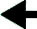 Case reports
	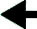 Protocols
Design	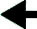 *In vitro* studies Animal studies
Language restrictions	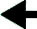 Not English or Chinese

*QoL, quality of life*.

### Data Sources and Search Strategies

Literature search was conducted comprehensively in four English databases (Web of Science, PubMed, Medline, and Cochrane Central) and three Chinese databases (China National Knowledge Infrastructure, Wanfang and Weipu). Additional references of relevant reviews or included articles were also manually screened. Articles published from inception to Apr 6, 2021, were included. The details regarding the search strategy are provided in Supplementary Table 1. Endnote X9 software was used to categorize and file all the references.

### Study Selection

Firstly, seven authors (HZ, ML, QL, ZL, YX, DY, and YL) screened retrieved articles and excluded irrelevant results according to titles and abstracts. Secondly, six authors (HZ, ML, QL, ZL, YX, and YL) conducted full-text review and obtained appropriate studies. They independently checked the included articles. The disagreements were resolved by discussion or consultation with two other authors (HH and COLU).

### Data Extraction

We used a standardized form to extract prespecified data elements from each study, including first author, year of publication, year of investigation, region, study design, age range or mean age if available, gender ratio (male/female), disease stage at diagnosis, sample source, sample size, duration of follow-up for the longitudinal studies, and main outcomes (economic burden and QoL). Two authors extracted data from obtained studies (ML and HZ), and disagreements were resolved by discussion or consultation with two other authors (HH and COLU). Excel 2013 was used to extract data and record.

### Quality Assessment

Study quality was assessed using the Effective Public Health Practice Project (EPHPP) tool, which included the following components: selection bias, study design, confounders, blinding, data collection methods, withdrawals and dropouts, intervention integrity, and data analysis. Two authors independently performed all quality ratings (HZ and ML), and disagreements were resolved by discussion or consultation with two other authors (HH and COLU).

## Results

### Study Selection

**Figure 1:**
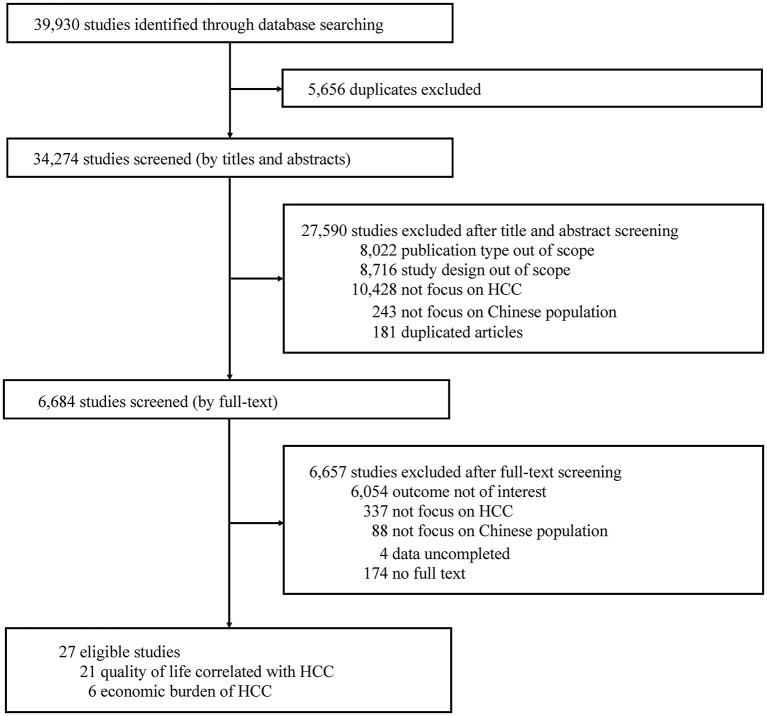
PRISMA flowchart of study selection.

We identified a total of 34,274 titles and abstracts by electronic searches, of which 6,684 were selected for full-text review (Figure 1). Twenty-seven publications were finally included reporting 6 studies in the economic burden and 21 in the QoL correlated with HCC.

### Assessment of Study Quality

The quality of the 6 economic burden studies was heterogeneous (Table 2). While 1 study relying on cancer registry claims data was regarded as having “strong” global rating (27), 2 studies based on hospital databases, social insurance information system, and national health insurance database were rated “moderate” (26, 29), and another 3 studies based on survey and hospital electronic health records were rated “weak” (25, 28, 30). A discrepancy occurred and resolved in raters, and the reasons were differences in interpretation of criteria.

**Table 2:** Quality assessment of identified studies.

**Study**	**Outcome**	**Component rating**						**Global rating**
		**Selection bias**	**Study design**	**Confounders**	**Blinding**	**Data collection methods**	**Withdrawals and dropouts**	
Che et al. (25)	Cost	Moderate	Weak	Weak	Moderate	Weak	NA	Weak
Hu et al. (26)	Cost	Moderate	Moderate	Weak	Moderate	Strong	NA	Moderate
Lang et al. (27)	Cost	Moderate	Moderate	Strong	Moderate	Strong	NA	Strong
Lei et al. (28)	Cost	Moderate	Weak	Weak	Moderate	Strong	NA	Weak
Nguang et al. (29)	Cost	Moderate	Moderate	Strong	Moderate	Strong	Weak	Moderate
Ran et al. (30)	Cost	Moderate	Weak	Weak	Moderate	Strong	NA	Weak
Fan et al. (31)	QoL	Moderate	Weak	Weak	Moderate	Strong	NA	Moderate
Fan et al. (32)	QoL	Moderate	Weak	Weak	Moderate	Strong	NA	Weak
Fielding et al. (33)	QoL	Moderate	Moderate	Strong	Moderate	Strong	Strong	Strong
Hsu et al. (34)	QoL	Moderate	Weak	Weak	Moderate	Strong	NA	Weak
Huang et al. (35)	QoL	Moderate	Weak	Weak	Moderate	Strong	NA	Weak
Li et al. (36)	QoL	Moderate	Moderate	Weak	Moderate	Strong	Strong	Moderate
Li et al. (37)	QoL	Moderate	Moderate	Weak	Moderate	Strong	Strong	Moderate
Li et al. (38)	QoL	Moderate	Moderate	Weak	Moderate	Strong	Strong	Moderate
Li et al. (39)	QoL	Moderate	Moderate	Weak	Moderate	Strong	Strong	Moderate
Jie et al. (40)	QoL	Moderate	Moderate	Strong	Moderate	Strong	Strong	Strong
Jie et al. (41)	QoL	Moderate	Moderate	Strong	Moderate	Strong	Strong	Strong
Qiao et al. (42)	QoL	Moderate	Weak	Weak	Moderate	Strong	NA	Weak
Qiu et al. (43)	QoL	Moderate	Weak	Weak	Moderate	Strong	NA	Weak
Shun et al. (44)	QoL	Moderate	Moderate	Strong	Moderate	Strong	Strong	Strong
Wei et al. (45)	QoL	Moderate	Weak	Weak	Moderate	Strong	NA	Weak
Wong et al. (46)	QoL	Moderate	Moderate	Strong	Moderate	Strong	Weak	Moderate
Wong et al. (47)	QoL	Moderate	Moderate	Strong	Moderate	Strong	Weak	Moderate
Yang et al. (48)	QoL	Moderate	Moderate	Weak	Moderate	Strong	Weak	Weak
Yeo et al. (49)	QoL	Strong	Moderate	Strong	Moderate	Strong	Strong	Strong
Zhang et al. (50)	QoL	Moderate	Moderate	Strong	Moderate	Strong	Strong	Strong
Zheng et al. (51)	QoL	Moderate	Weak	Weak	Moderate	Strong	NA	Weak

*QoL, quality of life; NA, not applicable*.

Six of 21 QoL studies were considered as having “strong” global rating, while 6 were considered as “moderate” and 9 were considered as “weak”. Data collection method was rated “strong” in all cases, since validated instruments were used for all evaluations. Studies with longer follow-up periods had major challenges in dropouts (46–48), which caused “weak” ratings in component of withdrawals and dropouts. Contrarily, study design was rated “weak” in cross-sectional studies (32, 34, 35, 42, 43, 45, 51) based on the definition of the EPHPP dictionary. Thirteen studies were rated “weak” in controlling for confounders because **<** 60% of relevant confounders were controlled or control of confounders not described (31, 32, 34–39, 42, 43, 45, 48, 51). A discrepancy occurred and resolved in raters, and the reasons were differences in interpretation of criteria.

### Economic Burden of HCC

#### Study Characteristics

HCC had a long-term and poor prognosis and the costs of HCC posed a huge economic burden for patients, household, and society worldwide. Six studies were included in this review after the data-extraction process (Figure 1): 3 were cross-sectional studies, 2 were cohort studies, and 1 was a case-control study. The characteristics of economic burden studies are summarized in Table 3.

**Table 3:** Characteristics of selected healthcare economic burden studies.

**Study**	**Perspective**	**Region, year of pricing**	**Study design**	**Sample source**	**Sample size**	**Main findings/Cost categories and costs**
Che et al. (25)	Patient	Yunnan, 2013	Cross-sectional study	Hospital-based	131 (of 940 HBV infected patients)	(1) Hospital costs accounted for the majority of total direct medical expenses. (2) Median estimated monthly household income: 4,000 CNY (IQR: 1,950, 9,750); Individual incomes: 2,000 CNY (IQR: 900, 3,000). (3) Insurance scheme [n (%)]: • No: 2 (1.5); UEM: 48 (36.6); URM: 27 (20.6); NRCM: 54 (41.2). (4) Estimated average annual direct costs for HCC patients was 33,044 CNY. (5) The percent of decrease in total direct expense (Total direct costs/household income per year median) after reimbursement was 36.1% in HCC group (33,044–17,273, 61.9–38.8%). (6) After reimbursement it could save 24% of patients not undergoing catastrophic health expenditure health in HCC groups.
Hu et al. (26)	Patient	Beijing and Guangzhou	Retrospective cohort Study	Hospital-based	596 CHB-related disease	(1) The total annual cost per patient for HCC was 6,615 USD in Beijing and 6,054 USD in Guangzhou. (2) Beijing: direct economic burden: 6,084 USD, indirect Economic Burden: 531 USD. (3) Guangzhou: direct economic burden: 5,472 USD, indirect Economic Burden: 582 USD.
Lang et al. (27)	Payer	Taiwan, 2002	Retrospective case-control study	Hospital-based	2873	(1) Group 1 (survived <1 year): 895 patients, average medical care costs: 206,573 TWD (6,259.7 USD, 2002), year 2002 value). (2) Group 2 (survived ≥1 year but died before the end of 2002): 735 patients, 237,032 TWD (7,182.7 USD, 2002). (3) Group 3 (still surviving by the end of 2002): 858 patients for initial phase, 140,403 TWD (4,254.6 USD, 2002); 1011 cases for continuous phase, 8,687 TWD (263.2 USD, 2002) per month. (4) For the average HCC patient, the 10-year lifetime cost was 418,554 TWD (12,683.4 USD, 2002).
Lei et al. (28)	Patient	13 Provinces, 2014	Cross-sectional study	Hospital-based	1196 (of 2223 liver cancer)	(1) HCC is the major pathological type of liver cancer. (2) Medical expenditure of HCC was 50,937 CNY, Non-medical expenditure was 4,592 CNY. Overall expenditure was 55,529 CNY. (3) Economic impact of overall expenditure on patients' family: • Expenditure of newly diagnosed course/Annual expenditure of illness of HCC: 48,150 CNY; • Self-reported predicted reimbursement ratio of HCC: 48.1%; • Out-of-pocket expenditure of HCC: 25,896 CNY; • Previous year household income: 56,536 CNY. (4) Self-reported degree of economic pressure of HCC: • Not at all (5.2%); • Somewhat but manageable (15.2%); • Heavy (30.5%); • Overwhelmed (49.1%).
Nguang et al. (29)	Payer	Taiwan, 2016	Retrospective cohort study	Hospital-based	5522	(1) Total healthcare expenditure (insurance payments) for treating HCC patients was approximately 92 million USD (92,269,551), including 53.4 million USD (58%) for hospital care and 38.7 million USD (42%) for outpatient and emergency department services. (2) Underlying comorbid conditions, liver transplants, hepatectomy, and trans arterial chemoembolization were associated with increased total cost, with liver transplants having the greatest impact over time.
Ran et al. (30)	Patient	Beijing, 2018	Cross-sectional study	Hospital-based	4174 hospitalizations	(1) Total hospitalization expenses: 158,320,500 CNY, average hospitalization expenses per time: 29,896.82 CNY (IQR:14,306.36, 50,567.79). (2) HCC was the second most expensive disease in this large tertiary hospital.

*UEM, the basic health insurance for urban employees medical scheme; URM, the basic health insurance for urban residents medical scheme; NRCM, the new rural cooperative medical scheme*.

#### Direct Costs

##### Medical Costs

Direct cost was divided into direct medical cost and direct nonmedical cost. Direct medical costs were generally collected through the hospital billing system (physician services, examination, medicine costs hospitalization charges) (52). Che et al. conducted a cross-sectional study to investigate the economic burden of patients at different stages of HBV-related liver diseases from December 2012 to June 2013 in Yunnan Province (25). Information of inpatients and outpatients were consecutively collected from medical records database and questionnaire in this study. A total of 131 patients with HCC were included whose monthly household and individual income was 4,000 CNY and 2,000 CNY respectively. For the financial burden of patients with HCC, the average annual direct costs were 33,044 CNY, while the average medical cost was 31,092 CNY.

Another hospital-based cross-sectional survey study covering 39 hospitals and 21 project sites in 13 provinces was carried out to investigate the economic burden of liver cancer in mainland China from 2012-2014 (28). Demographic and societal information, expenditures, and time loss due to clinical visits of 1,196 patients with HCC were investigated with the use of a structured questionnaire. The overall expenditure was 55,529 CNY, whereas medical expenditure was 50,937 CNY. The total expenditure of a newly diagnosed course of patients with HCC was 48,150 CNY, of which the reimbursed medical expenditures accounted for 48.1%. The out-of-pocket expenditure was estimated to be 25,896 CNY, which accounted for nearly 50% of the previous year's household income (56,536 CNY). In this study, 49.1% of patients with HCC considered the economic burden was overwhelming for them compared to only 19.7% of other patient groups who reported the same concerns (*P* < 0.001).

One retrospective cohort study conducted in Beijing and Guangzhou collected the data from the information system of community health center and a structured household questionnaire survey to investigate the economic impact of chronic HBV-related diseases and HCC was one of the conditions under investigation (26). In this study, the annual direct economic burden which referred to the economic resources for healthcare services utilized were 6,084 USD and 5,472 USD in Beijing and Guangzhou. Ran et al. analyzed the top 10 diseases in the total hospitalization cost of a tertiary hospital in Beijing between 2017–2018 (30). The results of the study showed that the average direct medical cost per visit for HCC was 29,896.82 CNY.

Nguang et al. retrospectively investigated the HCC-related burden in Taiwan (29). Data from national health insurance program research, the Longitudinal Health Insurance Database 2000 (LHID 2000), was extracted for analysis. From 1997 to 2012, 5,522 patients were newly diagnosed with HCC. The total healthcare expenditure for all patients with HCC was 92 million USD of which 58% was due to hospital cost, and 42% was due to outpatient and emergency department services. The average total cost per patient was 16,711 (±21,350) USD, of which inpatient care cost was 9,721 (±11,811) USD and outpatient cost was 6,989 (±14,726) USD.

Another prospective study was conducted to determine the lifetime cancer-related medical costs of patients with HCC in Taiwan by Lang et al. (27). The data of 2,592 patients with HCC was extracted from cancer registry statistics of patients with HCC and the claims data of Taipei Veterans General Hospital (TVGH) during 1999–2002. The costs of 3 HCC disease phases including the initial treatment phase (3 month period after diagnosis), continuous care phase and terminal phase (final 6 months prior to death) were applied to estimate the lifetime cost (53). For patients who survived <1 year (Group 1), all costs were calculated and the average cost was 206,573 TWD per person (6,259.7 USD, 2002). For patients in survived ≥ 1 year but died before the end of 2002 (Group 2), the data was used to derive the terminal cost and the cost was 237,032 TWD (7,182.7 USD, 2002). For patients who were still surviving by the end of 2002 (Group 3), the data was used to calculate the initial and monthly continuing costs. The cost was found to be 140,403 TWD (4,254.6 USD, 2002) at the initial phase, 8,687 TWD per month at the continuous phase (263.2 USD, 2002), and 418,554 TWD (12,683.4 USD, 2002) for a 10-year lifetime.

##### Non-medical Costs

Three studies explored the direct non-medical cost of patients with HCC by specific questionnaires. The non-medical costs in these studies normally referred to additional meals, additional nutrition supplements, transportation, accommodation and informal nursing worker fee due to illness. Che et al. reported the non-medical cost of patients with HCC in Yunnan province was 565 CNY annually (25). The average non-medical expenditure in 13 provinces in mainland China was 4,592 CNY per case (28).

#### Indirect Costs

The indirect costs were estimated from the social perspective in two studies in which the work loss of both patients and their families due to HCC were considered. Hu et al. reported that the average indirect economic burden of HCC in Beijing and Guangzhou were 531 USD and 582 USD (26). It was discovered by Lei et al. that the working day loss of patients and their caregivers due to HCC diagnosis was about 70.5 days, including 41.4 days for patients with HCC and 29.1 days for accompanying persons (28).

#### Health Insurance Payments

The impact of health insurance on patients' economic burden was also explored by two studies. Che et al. found almost all patients in Yunnan province were covered by at least one of the health insurance schemes (25): namely the New Rural Cooperative Medical Scheme (41.2%), the Urban Employee Basic Medical Insurance (36.6%), and the Urban Resident Basic Medical Insurance Scheme (20.6%). After reimbursement, the median percentage of decrease in annual total direct expense was 36.1% (11,928 CNY) in patients with HCC. In terms of the catastrophic health expenditure, health insurance could save more than 24% of patients with HCC not undergoing catastrophic health expenditure. Ran et al. also analyzed patients with medical insurance (30), and the results showed that during 2017–2018, medical insurance payments covered a total of 12,617.5 thousand CNY for patients with HCC.

### QoL Correlated With HCC

#### Study Characteristics

Of the 27 studies identified, 21 included outcomes on QoL effects: 11 of these were prospective cohort studies, 8 were cross-sectional studies, 1 was case-control study, and 1 was interrupted time series. The 21 studies encompassed a total of 3,060 HCC patients and were conducted in Shanghai (*n* = 1), Chongqing (*n* = 3), Hebei (*n* = 1), Guangxi (*n* = 1), Jiangxi (*n* = 1), Yunnan (*n* = 1), Hong Kong (*n* = 8), and Taiwan (*n* = 5). Summarized of characteristics of QoL studies are provided in Table 4.

**Table 4:** Characteristics of selected QoL studies.

**Study**	**Region**	**Study design**	**Sample source**	**Sample size**	**Measure(s)**	**QoL data**
**Measures of QoL in Chinese patients**						
Hsu et al. (34)	Taiwan	Cross-sectional study	Hospital-based	300	MNA-LF, MNA-SF, EORTC QLQ-C30 V3.0	(1) Both the long-form and short-form of the MNA performed better than GQL and GFS in predicting quality of life and functional status of patients with HCC . (2)The MNA is suitable for identifying the risk of deteriorating quality of life or functional status, in addition to identifying the risk of malnutrition, in patients with HCC.
Li et al. (36)	Hong Kong	Prospective cohort study	Hospital-based	472	EORTC QLQ-C30, EORTC QLQ-HCC18, C30 and HCC18 index-scores	(1) In multivariate analysis, independent prognostic HRQOL variables for OS were QLQ-C30 pain (HR 1.346 [1.092–1.661], *P* = 0.0055), QLQ-C30 physical functioning (HR 0.652 [0.495–0.860), *P* = 0.0024); QLQ-HCC18 pain (HR 1.382 [1.089–1.754], *P* = 0.0077) and QLQ-HCC18 fatigue (HR 1.441 [1.132–1.833], *P* = 0.0030). (2)C30 index-score (HR 2.143 [1.616–2.841], *P* < 0.0001) and HCC18 index-score (HR 1.957 [1.411–2.715], *P* < 0.0001) were highly significant factors for OS. (3)The median OS of patients with C30 index-score of 0–20, 21–40, 41–60, 61–100 were 16.4, 7.3, 3.1, 1.8 months respectively (*P* < 0.0001); while for HCC18 index-score: 16.4, 6.0, 2.8, 1.8 months respectively (*P* < 0.0001).
Yang et al. (48)	Yunnan	Interrupted time series	Hospital-based	114	EORTC QLQ-HCC18, FACT-Hep	(1)The internal consistency Cronbach's α were **>** 0.60 for all domains (exception of Jaundice 0.38), and all test-retest reliability coefficients were **>** 0.80. (2)Four out of eight domains had statistically significant changes with effect size standardized response mean ranging from 0.31 to 0.73.
**HCC compared with benign liver diseases and the general population**						
Fan et al. (32)	Taiwan	Cross-sectional study	Hospital-based	286	EORTC QLQ-C30, EORTC QLQ-HCC18, Brief IPQ, Jalowiec coping scale	(1) Patients with HCC had worse global QoL, physical, role, cognitive and social functioning, but better emotional functioning than the general population. (2)Cognitive representation was significant predictors of global QoL, physical functioning and emotional functioning. (3)Cognitive representation mediated the relationships between physical variables and global QoL, physical functioning and emotional functioning, but coping only mediated the relationship between cognitive representation and global QoL.
Wei et al. (45)	Guangxi	Cross-sectional study	Hospital-based	63	CD-RISC, EORTC QLQ-C30 V3.0, EORTC QLQ-HCC18	(1) The total health status score of QLQ-C30 was (56.61 ± 27.24) points, with lower quality of life scores and higher clinical symptoms scores. (2)Emotional functioning, social functioning, and general health status were positively correlated with the total score of psychological resilience (*r* = 0.382, *P* < 0.01; *r* = 0.324, *P* < 0.01; *r* = 0.383, *P* < 0.01). (3)Symptoms of fatigue, pain, fatigue, and nutritional changes were negatively correlated with the total score of psychological resilience (*r* = −0.303, *P* < 0.05; *r* = −0.286, *P* < 0.05; *r* = −0.360, *P* < 0.01; *r* = −0.259, *P* < 0.05).
Zhang et al. (50)	Hebei	Case-control study	Hospital-based	81 HCC 44 benign liver diseases	QOL-LC V2.0	(1) The scores of physical functioning (42 ± 10), symptom/side effect (41 ± 7), social functioning (28 ± 10) and total QoL (150 ± 24) of HCC group were lower than that of control group (*P* < 0.05). (2)The scores of physical functioning, symptom/side effect are negatively correlated with the way of case finding, TNM stage, Child-Pugh class, respectively. (3)The score of psychological functioning is positively correlated with age. (4)The score of social functioning is positively correlated with age, education level, income, and mode of payment, and negatively correlated with the way of case finding.
**Physical factors and symptoms associated with QoL**						
Fielding et al. (33)	Hong Kong	Prospective cohort study	Hospital-based	176 liver cancer 358 lung cancer	FACT-G, Visual analog: eating ability, eating appetite, eating enjoyment, self-care ability, and current health perception	(1)No association between QoL and survival in patients with liver cancer. (2)Less advanced cancer stage and better appetite were associated significantly with longer survival in patients with liver cancer.
Li et al. (37)	Hong Kong	Prospective cohort study	Hospital-based	445	EORTC QLQ-C30, EORTC QLQ-HCC18, C30 and HCC18 index-scores	(1)Significant correlations were found between IL-8 levels and EORTC QLQ-C30, QLQ-HCC18, C30, and HCC18 index-scores. (2)The strongest correlated factors were those reflective of constitutional symptoms, namely QLQ-C30 “appetite loss” (with Pearson's correlation coefficient, *r* = 0.322, *P* < 0.0001); QLQ-C30 “fatigue” (*r* = 0.311, *P* < 0.0001); QLQ-C30 “role functioning” (*r* = −0.305, *P* < 0.0001); QLQ-HCC18 “nutrition” (*r* = 0.317, *P* < 0.0001); and QLQ-HCC18 “fatigue” (*r* = 0.306, *P* < 0.0001). (3)Moderate but significant correlations were also observed with HCC18 index score (*r* = 0.321, *P* < 0.0001), and C30 index score (*r* = 0.306, *P* < 0.0001). (4)QoL factors were also significantly correlated with mIBI.
Li et al. (38)	Hong Kong	Prospective cohort study	Hospital-based	445	EORTC QLQ-C30, EORCT QLQ-HCC18, C30 and HCC18 index-scores	(1)Higher inflammatory states were significantly correlated with worse QoL. (2)For CRP and CRP/alb ratio, the QoL factors with higher correlations included C30 and HCC18 index-scores, certain QLQ-C30 domains and items (“physical functioning”, “role functioning”, “fatigue”, “pain”, “appetite loss”) and QLQ-HCC18 items (“fatigue”, “body image”, “nutrition” and “abdominal swelling”), where the Pearson's correlation coefficients were up to 0.416. (3)Multivariate analyses indicated that worse QoL factors were significantly correlated with worse scores in GPS, IBI and PI.
Li et al. (39)	Hong Kong	Prospective cohort study	Hospital-based	472	EORTC QLQ-C30, EORCT QLQ-HCC18, C30 and HCC18 index-scores	(1)After adjusting for clinical variables, significant correlations were found between QoL (QLQ-C30 and QLQ-HCC18) and dichotomized liver function variables (including Child-Pugh class, ALBI grade and the presence of ascites). (2)It was demonstrated that QoL had significant and potentially clinically important correlations with continuous liver function variables (albumin, bilirubin, ALP and albumin-to-ALP ratio), with the highest Spearman's rank correlation coefficient (rho) exceeding 0.4. (3)HCC18 and C30 index scores were also significantly correlated with these liver function variables. HCC18 index score, which had rho up to 0.37, generally performed better than C30 index score, which had rho up to 0.33.
Qiao et al. (42)	Shanghai	Cross-sectional study	Hospital-based	140	FACT-Hep	(1)The mean FACT-Hep scores were reduced significantly from TNM Stage I to Stage II, Stage IIIA, Stage IIIB group (687 ± 39.69 vs. 547 ± 42.57 vs. 387 ± 51.24 vs. 177 ± 71.44, *P* = 0.001). (2)Regarding the physical and emotional well-being subscales, scores decreased gradually from Stage I to Stage IIIB (*P* = 0.002 vs. Stage I; *P* = 0.032 vs. Stage II; *P* = 0.033 vs. Stage IIIA). (3)Mean FACT-Hep scores varied by Child-Pugh class, especially in the subscales of physical well-being, functional well-being and the hepatobiliary cancer (*P* = 0.001; *P* = 0.036; *P* = 0.032 vs. Child A). (4)For the social and family well-being subscale, only Child C scores were significantly lower as compared with Child A scores (*P* = 0.035). (5)For the subscales of functional well-being and hepatobiliary cancer, there were significant differences for Child A, B and C (*P* = 0.002 vs. Child A).
Wong et al. (46)	Hong Kong	Prospective cohort study	Hospital-based	253 liver cancer 334 lung cancer	FACT-G, MISS-Cog, ChPSQ-9, The single-item visual analog to assess eating appetite, optimism, and depression	(1)There were no differences in QoL, patient satisfaction, and psychosocial measures between the 2 cancer groups. (2)The patients' informational support from medical staff did not predict QoL, but all psychosocial factors emerged as a covariate of the satisfaction in predicting QoL; after controlling for sociodemographic and psychosocial variables, only satisfaction predicted QoL.
Wong et al. (47)	Hong Kong	Prospective cohort study	Hospital-based	253 liver cancer 250 breast cancer 334 lung cancer 242 nasopharyngeal cancer	FACT-G, Eating function: eating ability, eating appetite, eating enjoyment, Pain rating (visual analog), Depression (single item)	(1)Patients with liver cancer reported lower scores in total QoL, physical, functional, emotional, eating appetite, and depression than patients with nasopharyngeal cancer. (2)After controlling for sociodemographic and medical variables, pain, depression, and eating function significantly predicted overall QoL, physical, and functional well-being over time (all cancer). (3)Patients with liver cancer had a slightly decreased score in eating appetite, ability, and enjoyment.
Yeo et al. (49)	Hong Kong	Prospective cohort study	Hospital-based	233	EORTC QLQ-C30	(1)Significant independent predictors of shorter survival were advanced Okuda staging (*P* = 0.0030; HR = 2.058), high baseline total bilirubin (*P* = 0.0008; HR = 1.013) and worse QoL score in the appetite score domain (*P* = 0.0028; HR for 10 point increase = 1.070). (2)Patients who were entered into the chemotherapy trial (*P* = 0.0002; HR = 0.503), those who scored better in the physical functioning domain (*P* = 0.0034; HR for 10 point decrease = 0.911) and the role functioning domain (*P* = 0.0383; HR for 10 point decrease = 0.944) of the QoL questionnaire, were associated with longer survival.
**Demographic characteristics and psychological factors associated with QoL**						
Fan et al. (31)	Taiwan	Cross-sectional study	Hospital-based	33	Semistructured interview	(1)The impact of disease: HCC was associated with physical symptoms and psychosocial stress, as well as positive changes. (2)Illness perceptions: patients perceived HCC as a long-term and chronic disease that could not be cured but might be controlled. (3)Coping strategies: these included focusing on managing HCC and its symptoms, emotional responses, and leading a normal life.
Huang et al. (35)	Taiwan	Cross-sectional study	Hospital-based	77	BFI-T, PSQI-Taiwan Form, Depression subscale of the HADS	(1)Fatigue, sleep disturbance, and depression are positively interrelated and co-occur in patients with HCC. (2)Depression completely mediates the effects of sleep disturbance on fatigue.
Jie et al. (40)	Chongqing	Prospective cohort study	Hospital-based	218	EORTC QLQ-C30, Brief IPQ	(1)When comparing the patients in the disclosed group with the patients who were uninformed, the patients in the disclosed group had higher scores for global QoL at discharge (*P* = 0.013) and higher scores on understanding of their illness regarding illness perceptions (*P* = 0.022). (2)When comparing the patients in the ‘autonomy-satisfied' group with the patients whose desire for disclosure was not satisfied, the patients in the autonomy-satisfied group had better emotional functioning and better global QoL at discharge (*P* < 0.001 and *P* = 0.001, respectively). (3)Additionally, the patients in the autonomy-satisfied group had higher scores for personal control (*P* = 0.009) and lower scores for emotional reaction (*P* = 0.007) regarding illness perceptions, even after controlling for other confounding factors.
Jie et al. (41)	Chongqing	Prospective cohort study	Hospital-based	300	PCL-C, PTGI, EORTC QLQ-C30	(1)Compared with the uninformed group, patients in the disclosed group had lower scores for PTSS (*P* < 0.001), higher scores for PTG (*P* < 0.001), better emotional functioning (*P* < 0.001), and better global QoL (*P* = 0.006) at 1 month after discharge.
Qiu et al. (43)	Chongqing	Cross-sectional study	Hospital-based	220	EROTC QLQ-C30, Brief IPQ, SCSQ, SSRS	(1)The mean score of quality of life was obviously higher in the male patients than the female patients (*P* < 0.001). (2)In regard to illness perceptions, personal control (β = 1.707, *P* = 0.003), identity of symptoms (β = −1.315, *P* = 0.016) and illness comprehensibility (β = 1.489, *P* = 0.014) were significantly correlated to the global QoL.
Shun et al. (44)	Taiwan	Prospective cohort study	Hospital-based	104	SDS, HADS, SCNS-SF34	(1)Overall symptom distress decreased monthly, with the highest level before discharge. Compared with the elderly group, the young group had a significantly higher level of symptom distress (*P* = 0.024) but had a lower level of this 2 months after discharge. (2)Patients in the young group who were male (β = −14.24, *P* = 0.012) and single/widowed (β = −19.17, *P* = 0.033) and with higher levels of education (β = 1.38, *P* = 0.020), stage C (β = 30.21, *P* = 0.006), good functional status (β = 0.78, *P* = 0.024), and higher levels of symptom distress (β = 2.67, *P* < 0.0001) and anxiety (β = 6.03, *P* < 0.0001) had higher levels of overall unmet supportive care needs. (3)For the elderly group, those patients with recent diagnosis status (β = −14.08, *P* = 0.008), portal vein thrombosis (β = 31.32, *P* = 0.011), and higher levels of symptom distress (β = 2.09, *P* = 0.003), anxiety (β = 4.60, *P* < 0.0001), and depression (β = 2.71, *P* = 0.007) had higher levels of overall unmet care needs.
Zheng et al. (51)	Jiangxi	Cross-sectional study	Hospital-based	166	EORTC QLQ-HCC18, HCC18 index-score, CES-D, RSES, LOT-R	(1)Scores of CES-D, RSES, and LOT-R were (17.34 ± 2.25), (29.59 ± 4.67), and (29.78 ± 3.14), respectively. (2)EORTC QLQ-HCC18, CES-D, RSES, and LOT-R were correlated with scores in various fields (*P* < 0. 05). (3)In the influence path of QoL on depressive symptoms, RSES regulation belonged to a complete mediation model and LOT-R regulation belonged to partial intermediary model.

#### Generic, Disease-Specific and Supplementary Measures of QoL in Chinese Patients

The European Organization for Research and Treatment for Cancer Quality of Life Questionnaire Core-30 (EORTC QLQ-C30) (*n* = 11) and the Functional Assessment of Cancer Treatment-General (FACT-G) (*n* = 3) were the 2 most commonly used measures to assess generic QoL. The EORTC QLQ-C30 reported 5 functional domains (physical, role, emotional, cognitive and social functioning), 8 symptom domains, 1 financial problem and 1 global health status (54). The FACT-G included 4 subscales for physical, social/family, emotional and functional well-being (55).

The European Organization for Research and Treatment of Cancer Quality of Life Questionnaire Core-18 (EORTC QLQ-HCC18) (*n* = 8), the Functional Assessment of Cancer Treatment-Hepatobiliary (FACT-Hep) (*n* = 2), and the quality of life-liver cancer (QOL-LC) (*n* = 1) were the 3 standardized instruments to assess disease-specific QoL. The EORTC QLQ-HCC18 assessed 8 symptom domains for fatigue, body image, jaundice, nutrition, pain, fever, abdominal swelling and sex life, which was primarily proposed for patients with HCC (56). The FACT-Hep was used widely for liver cancer-specific assessment, consisting of FACT-G and newly validated hepatobiliary subscale (57). The Chinese versions of both of these tools have demonstrated good validity, reliability, and responsiveness (48, 58). For Chinese patients diagnosed with liver cancer, the QOL-LC was specifically developed, and the disease-specific items were evaluated in terms of the symptom/side effect subscale (59).

In addition to conventional assessment instruments mentioned above, 2 original studies reported the development of supplementary measures for Chinese patients with HCC, which included QLQ-C30, QLQ-HCC18 index scores and Mini Nutritional Assessment (MNA) (34, 36). C30 and HCC18 index scores could represent all domains of QLQ-C30 and HCC18, respectively (36). The mathematical formulas were presented as follows:


$$\begin{array}{l} C30\;index\;score=[(100-global\;QoL)+(100-physical\;functioning)\;\\ \;+\;(100-role\;fuctioning)+(100-emotional\;functioning)\;\\ \;+(100-cognitive\;functioning)+(100-social\;functioning)\;\\ \;+fatigue+nausea\;and\;vomiting+pain+dyspnoea+insomnia\;\\ \;+appetite\;loss+constipation+diarrhea+financial\;difficulties]/15;\;\\ \;HCC18\;index\;score=\;(fatigue+body\;image+jaundice+nutrition+pain\;\\ \;+fever+abdominal\;swelling\;+sex\;life)/8 \end{array}$$

(36).

The index scores had more advantages in clinical practice, since they converted complex QoL data to more straightforward tools. The MNA, a nutritional assessment tool, performed better than global functional status and global QoL of the EORTC QLQ-C30 to predict functional status and QoL in Chinese patients with HCC (34).

#### QoL in Chinese Patients With HCC Compared With Benign Liver Diseases and the General Population

##### HCC Compared With Benign Liver Diseases

Patients with HCC had lower physical functioning (QOL-LC score: 42 ± 10), symptoms/side effects (41 ± 7), social functioning (28 ± 10) and global QoL (150 ± 24) than patients with benign liver diseases (*P* < 0.05), but showed no difference in psychological functioning (39 ± 10 vs. 40 ± 10; *P* > 0.05) and self-assessment (70 ± 15 vs. 75 ± 15; *P* > 0.05) (50).

##### HCC Compared With the General Population

Patients with HCC had worse QoL than the general population, especially in global QoL (32, 45), physical (32, 45), role (32), cognitive (32), social functioning (32, 45), pain (45), insomnia (45), appetite loss (45), and financial difficulties (45). Conversely, patients reported better scores in emotional functioning (*P* < 0.05 for all) (32).

The EORTC QLQ-C30 was applied in 11 of 21 studies to measure QoL, but means and standard deviations were only provided in 6 studies (34, 36, 41, 43, 45, 49). We compared the pooled means and standard deviations in baseline of the 6 studies (total HCC cases, 1,588) with the norms which provided by EORTC Group for the general population and patients with heterogeneous cancer (Table 5) (60). Patients with HCC had worse global QoL (*t* = −6.78; *P* < 0.01), physical functioning (*t* = −4.79; *P* < 0.01), cognitive functioning (*t* = −4.766; *P* < 0.01), social functioning (*t* = −6.798; *P* < 0.01), fatigue (*t* = 5.588, *P* < 0.05), appetite loss (*t* = 3.792; *P* < 0.05) and financial difficulties (*t* = 5.149; *P* < 0.05) than the general population. Additionally, patients with HCC had worse cognitive functioning (*t* = −3.049; *P* < 0.05), but better role functioning (*t* = 3.44; *P* < 0.05) and emotional functioning (*t* = 2.546; *P* < 0.05) than cancer patients.

**Table 5:** The pooled data and norms of the EORTC QLQ-C30 subscales.

	**Pooled data**	**Norms, general population*^a^***	**Norms, heterogeneous cancer*^a^***
N	1,588	7,802	23,553
Global quality of life	57.1 (5.5)	71.2 (22.4)	61.3 (24.2)
Physical functioning	77.6 (6.7)	89.8 (16.2)	76.7 (23.2)
Role functioning	81.2 (8.2)	84.7 (25.4)	70.5 (32.8)
Emotional functioning	74.9 (3.6)	76.3 (22.8)	71.4 (24.2)
Cognitive functioning	76.4 (5.4)	86.1 (20)	82.6 (21.9)
Social functioning	68.5 (7.4)	87.5 (22.9)	75.0 (29.1)
Fatigue	36.4 (4.4)	24.1 (24)	34.6 (27.8)
Nausea/vomiting	8.9 (2.5)	3.7 (11.7)	9.1 (19)
Pain	26.4 (9.5)	20.9 (27.6)	27.0 (29.9)
Dyspnoea	20.3 (8.2)	11.8 (22.8)	21.0 (28.4)
Insomnia	27.5 (9.4)	21.8 (29.7)	28.9 (31.9)
Appetite loss	20.8 (8.3)	6.7 (18.3)	21.1 (31.3)
Constipation	13.4 (3.8)	6.7 (18.4)	17.5 (28.4)
Diarrhea	12.7 (3.2)	7.0 (18)	9.0 (20.3)
Financial difficulties	40.8 (10.5)	9.5 (23.3)	16.3 (28.1)

*Means and standard deviations shown*.

a *Data from Scott et al. (60)*.

#### Physical Factors and Symptoms Associated With QoL in Chinese Patients

##### Physical Factors Associated With QoL

Better liver function was significantly correlated with better QoL. Patients with better Child–Pugh classification had better QoL, mainly in terms of higher physical well-being, functional well-being and fewer hepatobiliary symptoms (*P* < 0.05) (39, 42, 50). Similarly, patients with better ALBI grade or the presence of ascites had higher physical functioning or worse abdominal swelling respectively (*P* < 0.001) (39). In addition to dichotomized liver function variables, lower serum bilirubin levels, higher albumin levels, lower alkaline phosphatase (ALP) levels, better albumin-to-ALP ratios were correlated with better QoL (39).

Tumor stages were negatively related to QoL. The mean FACT-Hep scores of Chinese patients with HCC were markedly decreased with the increasing TNM Stages (Stage I was 687 ± 39.69 vs. Stage II 547 ± 42.57 vs. Stage IIIA 387 ± 51.24 vs. Stage IIIB 177 ± 71.44; *P* = 0.001) (42). The most impaired subscales of QoL were physical, emotional well-being (measured by FACT-Hep; *P* < 0.05) and symptom/side effect (measured by QOL-LC; *P* < 0.05) (42, 50).

There were also significant negative correlations between inflammatory states and QoL. Patients with higher IL-8 levels suffered worse QoL, especially in constitutional symptoms, namely worse role functioning, appetite loss, fatigue, and nutrition (*P* < 0.0001) (37). For C-reactive protein (CRP) and CRP-to-albumin ratio, the QoL factors with higher negative correlations included physical and role functioning, as well as with higher positive correlations included fatigue, pain, appetite loss, body image, nutrition, and abdominal swelling (*P* < 0.0001 for all) (38). Worse QoL factors were also significantly associated with worse scores in Inflammation-Based Index, Glasgow Prognostic Score, and Prognostic index (37, 38).

Furthermore, QoL was considered as a significant predictor for survival time (36, 49). Yeo et al. indicated that significant independent predictors for survival were QLQ-C30 physical functioning (HR 0.911 [0.856-0.969]; *P* = 0.0034), role functioning (HR 0.944 [0.894-0.996]; *P* = 0.0383) and appetite loss (HR 1.070 [1.023–1.118]; *P* = 0.0028) in patients with unresectable HCC (49), whereas the results from Li et al. reported that independent prognostic QoL factors for survival consisted of QLQ-C30 physical functioning (HR 0.652 [0.495–0.860]; *P* = 0.0024), pain (HR 1.346 [1.092–1.661]; *P* = 0.0055), QLQ-HCC18 fatigue (HR 1.441 [1.132–1.833]; *P* = 0.0030), pain (HR 1.382 [1.089–1.754]; *P* = 0.0077) in patients affected by any stage of HCC (36).

##### Symptoms Associated With QoL

Severe symptoms were significantly associated with worse QoL, including appetite loss and pain (46). It was proved that worse eating appetite (*P* < 0.001) and higher reported pain (*P* < 0.001) predicted lower FACT-G total scores (worse QoL) (46). Moreover, eating ability and performance status were correlated positively with patient QoL (32, 47).

#### Demographic Characteristics and Psychological Factors Associated With QoL in Chinese Patients

##### Demographic Characteristics Associated With QoL

Older patients had better QoL (44, 50), mainly in terms of psychological functioning and social functioning (*P* < 0.05) (50). The female gender was associated with worse QoL (QLQ-C30 score for female patients was 41.36 ± 24.06 vs. 55.74 ± 22.03 for male patients; *P* < 0.001) (43). Higher education levels were correlated with better social functioning (*P* < 0.05) (50). Furthermore, income and mode of payment were correlated positively with social functioning, while the way of case finding was correlated negatively with physical and social functioning and correlated positively with symptoms/side effects (*P* < 0.05) (50).

##### Psychological Factors Associated With QoL

Depression was correlated negatively with QoL (47). One study showed positive correlations among depression, sleep disturbance and fatigue. These symptoms occurred together in HCC patients. Depression had complete mediating effects for the impact of sleep disturbance on fatigue (35). Meanwhile, a recent study showed that the EORTC QLQ-HCC18 and Center for Epidemiological Survey, Depression Scale (CES-D) for HCC patients were correlated with scores in various fields (*P* < 0.05), and self-esteem and optimism completely and partially mediated the effects of QoL on depressive symptoms respectively (51), which suggested that psychological intervention to strengthen the patient′s self-esteem and optimism could be helpful to reduce the symptoms of depression in patients.

Moreover, QoL and depression were significantly associated with care needs. This was supported by evidence that patients with good functional status and higher levels of symptom distress, anxiety and depression had higher levels of overall unmet care needs (*P* < 0.05) (44).

Psychological resilience and QoL were positively interrelated. Emotional, social functioning, and global QoL were positively correlated with psychological resilience (*r* = 0.382, *P* < 0.01; *r* = 0.324, *P* < 0.01; *r* = 0.383, *P* < 0.01) (45). Symptoms of fatigue, pain, fatigue, and nutritional changes were negatively correlated with psychological resilience (*r* = −0.303, *P* < 0.05; *r* = −0.286, *P* < 0.05; *r* = −0.360, *P* < 0.01; *r* = −0.259, *P* < 0.05) (45). Clinically, improving psychological resilience of HCC patients could promote symptom management and improve QoL.

Illness perception and coping were significantly correlated with QoL. Patients who had positive illness perceptions had better QoL, while patients who made more emotion-oriented coping reported worse QoL (31, 32). Furthermore, personal control (β = 1.707, *P* = 0.003), identity of symptoms (β = −1.315, *P* = 0.016) and illness comprehensibility (β = 1.489, *P* = 0.014) regarding illness perceptions were greatly correlated with global QoL (43).

Additionally, diagnosis disclosure and patient autonomy had impacts on illness perceptions and QoL. Patients who were aware of cancer diagnosis had better illness comprehensibility (*P* = 0.022) and global QoL (*P* = 0.013) (40). Autonomy-satisfied patients had lower emotional reaction (*P* = 0.007) and better personal control (*P* = 0.009), and had better global QoL (*P* = 0.001), emotional functioning (*P* < 0.001) (40). Consequently, the desires of patients for autonomy in regard to their diagnosis disclosure should be satisfied. Meanwhile, diagnosis disclosure also correlated to less severe posttraumatic stress symptoms (*P* < 0.001), greater posttraumatic growth (*P* < 0.001), better global QoL (*P* = 0.006), and better emotional functioning (*P* < 0.001) (41). Besides, patients who satisfied with medical services had better QoL (46).

## Discussion

This systematic review provided a landscape of the burden caused by HCC in greater China in terms of economic and QoL dimensions from database inception to Apr 6, 2021. The economic impacts on patients with HCC have been receiving rising attention worldwide in the past decades (8, 61–63). Nevertheless, few studies have reported the economic burden of patients with HCC in greater China, and only 6 studies have been identified and included in the present review. As far as the study region was concerned, no publication investigated the economic burden of patients with HCC at a national level. Despite the 2 studies that analyzed the expenditure of HCC in one province or city (26, 28), other studies only focused on a single area, including Taiwan (27, 29), Yunnan province (25) and Beijing (30).

The research methodologies and data sources employed in the included studies were heterogeneous. Two cross-sectional designs and one cohort design were adopted using questionnaires to explore more specific individual expenditures (25, 26, 28). The data of 3 other studies were drawn from the expenditure data of different electronic databases, including the national health insurance database, cancer registry claims data and hospital health records. Such research designs only provided the information of patients' direct medical costs. Non-medical costs and indirect costs, such as supportive services, loss of productivity and family burden, were not considered individually. Although direct hospitalization costs caused huge economic burden for patients with HCC, numerous studies have shown that in addition to the direct medical costs, the economic burden on the family caused by the disease was also extremely significant (64, 65). Therefore, the studies that analyzed the hospitalization data from database could not fully explain the other costs that HCC patients and their families had to bear. In this review, 3 studies that employed questionnaire design reported the economic burden of patients in greater China. One cohort study analyzed both the direct medical costs and indirect costs, and 2 cross-sectional studies investigated the medical costs, non-medical costs and family/household health expenditure of patients with HCC. In 2009, Hu et al. reported that the annual economic burden (direct and indirect cost) of patients with HCC in Beijing and Guangzhou were 6,615 USD (45,180.5 CNY, 2009), and 6,054 USD (41,348.8 CNY, 2009), where the direct cost accounted for 92.0% [6,084 USD (41,553.72 CNY, 2009)] and 90.4% [5,472 USD (37,373.76 CNY, 2009)] (26). The study conducted by Che et al. in Yunnan province between 2012 and 2013 showed that the average annual direct costs for patients with HCC were 33,044 CNY of which the total direct costs/household income per year was 61.9%. These results revealed the big economic impact of HCC on patients and their families (25). Another research reported that the average total expenditure per HCC patient was 55,529 CNY during 2012-2014 based on the 13 provinces in mainland China, including 50,937 CNY of medical expenditure and 4,592 CNY of non-medical expenditure (28). Based on current studies in greater China, HCC imposed a substantial economic burden on patients. The results also showed that health insurance reimbursement could alleviate patients' health care financial burden with high levels of household economic status. The patients who lived in first-line cities might have a higher economic burden than patients who lived in other regions, and the cost of patients with HCC from hospitals and communities also varied greatly, prompting the need for further investigation in the future.

Almost all studies in this review reported the annual direct medical expenditure of HCC. Studies in the US reported that the median overall direct costs for per patient with HCC were 176,456 USD (66), and the annual estimates of lost productivity were 3,553 USD per patient (63). In Australia, the total health expenditure attributed to HCC in 2019–2020 was estimated to be 31,775 USD per patient with HCC, while the total cost of HCC inpatient admissions was 26,438 USD per patient (67). The cost of HCC in Japan was calculated to be 607.2 billion JPY in 2014 (68). It was difficult to directly compare the costs of HCC among different studies due to different regions, health systems, calculation methodologies, the currency and year of calculating prices. Since greater China still had a relatively lower average household income than these developed countries, the economic burden of HCC patients in greater China was still large.

Concerning the QoL burden, the EORTC QLQ-C30 and the FACT-G were the most commonly used measures to assess cancer-specific QoL in patients diagnosed as HCC. The EORTC QLQ-HCC18 and the FACT-Hep supplemented the EORTC QLQ-C30 and the FACT-G, respectively, as disease-specific modules for the assessment of patients with HCC. The Chinese versions of these 4 measures showed good validity and reliability (48, 58, 69, 70), and were suitable for use in Chinese patients. The QOL-LC was also appropriate for Chinese patients due to its cultural sensitivity. We further identified 3 supplementary measures of QoL for use involving Chinese patients. C30 and HCC18 index scores were developed to summarize and analyse raw QoL data to render QLQ-C30 and HCC18 more communicable and meaningful (36). The MNA was suitable to identify the malnutrition and deteriorating QoL for Chinese patients (34). These tools could serve potentially as standardized measures for QoL research regarding patients with HCC in the future.

Compared with medical indices such as functional index, survival rate and mortality rate, the scores of QoL could be more informative as it comprehensively covered all aspects of performance and health status (71). The present review suggested that compared with the general population, Chinese patients with HCC had poor QoL in terms of physical, cognitive functioning and severe symptoms, consistent with the previous results based on the patients with HCC across the globe (72). However, Chinese patients also had worse social relationships, different from the global result (72). Additionally, Chinese patients with HCC had poorer QoL than patients who had benign liver diseases, especially social and physical aspects. Side effects of treatments and severe symptoms might cause impaired physical functioning, especially weight loss, pain, digestive problem, diarrhea and fever.

The physical and psychosocial predictors for QoL in Chinese patients with HCC were identified in this review. Regarding the physical variables, patients who had a better liver function, early tumor stage, less severe inflammatory states and symptoms, and better eating ability and performance status had better QoL. Psychosocial factors such as better psychosocial resilience, positive illness perceptions, diagnosis disclosure, patient autonomy, and satisfaction of medical services predicted better QoL, whereas depression and emotion-oriented coping indicated worse QoL. The findings suggested that physicians and patients could improve these physical and psychosocial factors to promote QoL correlated with HCC in daily clinical practice.

This systematic review provided the most up-to-date and in-depth overview of the available data about the cost and QoL of HCC in greater China. It revealed the substantial economic and QoL burden of HCC in the region. The findings can contribute to gauge whether health resource allocations match the burden of HCC in greater China and supply useful information for evaluating the population's health status. The burden of HCC measures can also provide significant insights into clinical gaps, leading to further research on the causes of the HCC burden and their resolutions. In addition, as we make great efforts to measure and improve our knowledge of the HCC burden, we may have an opportunity to address the existing methodological challenges to investigate this field further.

However, several limitations of this review can be addressed in future studies. Firstly, limited research provided information on HCC disease staging or progression related to the frequency of clinical services and hospitalizations. More comprehensive health-related costs may be demonstrated by taking into account further disease information. Secondly, differences in the results reported might be a consequence of differences in methodologies and tools for measurement. Thirdly, only a few studies provided the baseline means and standard deviations of QoL data, resulting in limitations regarding pooled data and conduction of meta-analysis. Fourthly, norms of EORTC for the general population were based on individuals from multiple nations except China and might not be representative of Chinese populations. However, a growing body of research is now exploring the applicability of the applications of EORTC in greater China, and there will be more evident in the future to verify reliability and validity of EORTC in Chinese populations. Lastly, living standards in greater China have improved substantially over the past few decades, and improvements in socioeconomic structure, health system, clinical treatment, patient education, and employment support may also affect the disease economic costs and QoL for HCC patients. The specific and quantitative relationship between living standards and disease burden requires further investigation.

## Conclusion

In summary, HCC has bought a significant economic burden to patients and their families in greater China. HCC also negatively impacted QoL in Chinese patients, especially in physical, cognitive, social functioning and severe symptoms. As the data about the economic and QoL burden of HCC in greater China is lacking, more investigations are needed to provide a deeper interpretation of the disease burden of patients with HCC in greater China in recent years. Moreover, further studies should focus on the disease-related economic impacts on both patients and their families, the effects of physical factors and psychological factors on QoL, the interactions between physical factors and psychological factors, and other predictors that can improve patients' QoL with HCC in greater China.

## Data Availability Statement

The original contributions presented in the study are included in the article/Supplementary Material, further inquiries can be directed to the corresponding author/s.

## Author Contributions

HZ, CU, and HH conceived the research project and coordinated the contributors. HZ, ML, QL, ZL, YX, DY, and YL participated in the study selection. HZ and ML extracted, analyzed and interpreted the data, and drafted the manuscript. HH and CU made critical revisions to the manuscript and supervised the study. All the authors reviewed and approved the final vision.

## Funding

This research was partially supported by a grant from the University of Macau (MYRG2020-00230-ICMS).

## Conflict of Interest

The authors declare that the research was conducted in the absence of any commercial or financial relationships that could be construed as a potential conflict of interest.

## Publisher's Note

All claims expressed in this article are solely those of the authors and do not necessarily represent those of their affiliated organizations, or those of the publisher, the editors and the reviewers. Any product that may be evaluated in this article, or claim that may be made by its manufacturer, is not guaranteed or endorsed by the publisher.
