# Supplementary material for: Economic Burden and Quality of Life of Hepatocellular Carcinoma in Greater China: A Systematic Review
Source: Front Public Health. 2022 Apr 21;10:801981. doi: 10.3389/fpubh.2022.801981 (PMC9068962; doi:10.3389/fpubh.2022.801981)
Supplement: Supplementary file 1 [file Table_1.DOCX]

Supplementary Material

**Supplementary Table 1.** Search strategy for epidemiologic, humanistic and economic burden of HCC in greater China.

|  | Search terms |
| --- | --- |
| Epidemiology | |
| 1 | (liver OR hepat*) ti,ab,kw. |
| 2 | (neoplas* OR cancer* OR tumor* OR tumour* OR carcinoma* OR oncolog* OR malign*) |
| 3 | *epidemiology/ OR *incidence/ OR *prevalence/ OR *morbidity/ OR *comorbidity/ |
| 4 | China OR Chinese OR Hong Kong OR Macau OR Taiwan |
| 5 | 1 AND 2 AND 3 AND 4 |
| Burden of disease | |
| 1 | liver OR hepat* |
| 2 | (neoplas* OR cancer* OR tumor* OR tumour* OR carcinoma* OR oncolog* OR malign*) |
| 3 | (disease progression OR disease course).mp. and (hepatitis C OR hep C OR HCV/ OR hepatitis B OR hep B OR HBV/ OR non?alcoholic steatohepatitis/ OR NASH/ OR NAFLD/ OR fatty liver disease/ OR liver cirrhosis/ OR *hepatocellular cancer/ OR liver cancer/ OR *steatosis/ OR *lobular inflammation/ OR *hepatocellular ballooning/ OR liver fibrosis/ OR liver decomposition/ OR non-liver cancer$/) [mp=ti, ab, hw, tn, ot, dm, mf, dv, kw, fx, tc, id, tm, ct, nm, kf, px, rx, ui, sy] |
| 4 | Quality of life/ OR quality of life.mp. OR quality adjusted life years/ OR QoL/ OR HRQoL/ OR health related quality of life/ OR patient reported outcome*.mp. OR quality adjusted life years/ OR QALY/ OR disability adjusted life years.mp. OR DALY/ OR productivity/ OR absenteeism/ OR presenteeism/ OR EQ#5D/ OR utility.mp. [mp=ti, ab, hw, tn, ot, dm, mf, dv, kw, fx, tc, id, tm, ct, nm, kf, px, rx, ui, sy] |
| 5 | economic$/ OR pharmacoeconomic$/ OR *cost benefit analysis/ OR *cost effectiveness analysis/ OR cost of illness/ OR cost control/ OR *health economics/ OR hospital cost/ OR cost minimization analysis/ OR (cost adj estimate$).mp. OR (cost adj variable$).mp. OR (unit adj cost$).mp. OR (disease model OR health?care utilisation OR registry OR database).mp. |
| 6 | *cost of illness/ OR *cost of management/ OR health care cost$/ OR *direct cost$/ OR *indirect cost$/ OR *drug cost$/ OR *treatment cost$/ OR (hospitali#ation.mp. adj cost$/) OR (health?care.mp. adj expenditure/) OR (health?care.mp. adj cost$/) OR (health?care resource.mp. adj utili#ation/) [mp=ti, ab, hw, tn, ot, dm, mf, dv, kw, fx, tc, id, tm, ct, nm, kf, px, rx, ui, sy] |
| 7 | China OR Chinese OR Hong Kong OR Macau OR Taiwan |
| 8 | 1 AND 2 AND 3 AND 7 |
| 9 | 1 AND 2 AND 4 AND 7 |
| 10 | 1 AND 2 AND 5 AND 7 |
| 11 | 1 AND 2 AND 6 AND 7 |
| 12 | 8 OR 9 OR 10 OR 11 |
